# Recommendations for breast cancer screening in Brazil, from the Brazilian College of Radiology and Diagnostic Imaging, the Brazilian Society of Mastology, and the Brazilian Federation of Gynecology and Obstetrics Associations

**DOI:** 10.1590/0100-3984.2023.0064-en

**Published:** 2023

**Authors:** Linei Augusta Brolini Delle Urban, Luciano Fernandes Chala, Ivie Braga de Paula, Selma di Pace Bauab, Marcela Brisighelli Schaefer, Ana Lúcia Kefalás Oliveira, Carlos Shimizu, Tatiane Mendes Gonçalves de Oliveira, Paula de Camargo Moraes, Beatriz Medicis Maranhão Miranda, Flávia Engel Aduan, Salete de Jesus Fonseca Rego, Ellyete de Oliveira Canella, Henrique Lima Couto, Gustavo Machado Badan, José Luis Esteves Francisco, Thaís Paiva Moraes, Rosangela Requi Jakubiak, João Emílio Peixoto

**Affiliations:** 1 Members of the National Mammography Commission, Representatives of the Colégio Brasileiro de Radiologia e Diagnóstico por Imagem (CBR), São Paulo, SP, Brazil; 2 Coordinator of the National Mammography Commission of the Colégio Brasileiro de Radiologia e Diagnóstico por Imagem (CBR), São Paulo, SP, Brazil; 3 Members of the National Mammography Commission, Representatives of the Sociedade Brasileira de Mastologia (SBM), Rio de Janeiro, RJ, Brazil; 4 Members of the National Mammography Commission, Representatives of the Federação Brasileira das Associações de Ginecologia e Obstetrícia (FEBRASGO), Rio de Janeiro, RJ, Brazil

**Keywords:** Breast neoplasms/diagnostic imaging, Early detection of cancer, Mammography, Ultrasonography, Magnetic resonance imaging, Practice guideline, Neoplasias da mama/diagnóstico por imagem, Detecção precoce de câncer, Mamografia, Ultrassonografia, Ressonância magnética, Guia de prática clínica

## Abstract

**Objective:**

To present an update of the recommendations of the Brazilian College of Radiology and Diagnostic Imaging, the Brazilian Society of Mastology, and the Brazilian Federation of Gynecology and Obstetrics Associations for breast cancer screening in Brazil.

**Materials and Methods:**

Scientific evidence published between January 2012 and July 2022 was gathered from the following databases: Medline (PubMed); Excerpta Medica (Embase); Cochrane Library; Ebsco; Cumulative Index to Nursing and Allied Health Literature (Cinahl); and Latin-American and Caribbean Health Sciences Literature (Lilacs). Recommendations were based on that evidence and were arrived at by consensus of a joint committee of experts from the three entities.

Recommendations: Annual mammographic screening is recommended for women between 40 and 74 years of age. For women at or above the age of 75, screening should be reserved for those with a life expectancy greater than seven years. Women at higher than average risk are considered by category: those with dense breasts; those with a personal history of atypical lobular hyperplasia, classical lobular carcinoma in situ, or atypical ductal hyperplasia; those previously treated for breast cancer; those having undergone thoracic radiotherapy before age 30; and those with a relevant genetic mutation or a strong family history. The benefits of complementary screening are also addressed according to the subcategories above. The use of tomosynthesis, which is an evolved form of mammography, should be considered in screening, whenever accessible and available.

## Abstract (pt)

**Objetivo:**

Apresentar a atualização das recomendações do Colégio Brasileiro de Radiologia e Diagnóstico por Imagem, da Sociedade Brasileira de Mastologia e da Federação Brasileira das Associações de Ginecologia e Obstetrícia para o rastreamento do câncer de mama no Brasil.

**Materiais e Métodos:**

Foram feitas buscas das evidências científicas publicadas nas bases Medline (PubMed), Excerpta Medica (Embase), Cochrane Library, Ebsco, Cinahl e Lilacs, entre janeiro de 2012 e julho de 2022. As recomendações foram baseadas nessas evidências, mediante consenso da comissão de especialistas das três entidades.

Recomendações: O rastreamento mamográfico anual é recomendado para as mulheres de risco habitual entre 40 e 74 anos. Acima de 75 anos deve ser reservado para as que tenham expectativa de vida maior que sete anos. Mulheres com risco maior que o habitual, entre elas as com mamas densas, com história pessoal de hiperplasia lobular atípica, carcinoma lobular *in situ* clássico, hiperplasia ductal atípica, tratamento de câncer de mama ou de irradiação no tórax antes dos 30 anos, ou ainda portadoras de mutação genética ou com forte história familiar, se beneficiam do rastreamento complementar, sendo consideradas de forma individualizada. A tomossíntese é uma evolução da mamografia e deve ser considerada no rastreamento, sempre que acessível e disponível.

## INTRODUCTION

In 2021, breast cancer came to be the most commonly diagnosed type of cancer and the leading cause of death among women worldwide^(1)^. In Brazil, there were an estimated 73,610 new cases of breast cancer in 2023, which translates to an adjusted incidence rate of 41.89 cases per 100,000 women^(1)^. Screening is an effective strategy to detect the disease at an early stage and reduce its mortality. In addition, early diagnosis allows a greater range of therapeutic options and reduces treatment morbidity^(2-4)^.

In 2012 and 2017, the *Colégio Brasileiro de Radiologia e Diagnóstico por Imagem* (CBR, Brazilian College of Radiology and Diagnostic Imaging), the *Sociedade Brasileira de Mastologia* (SBM, Brazilian Society of Mastology), and the *Federação Brasileira das Associações de Ginecologia e Obstetrícia* (FEBRASGO, Brazilian Federation of Gynecology and Obstetrics Associations) published recommendations for breast cancer screening^(5,6)^, under the auspices of the National Mammography Commission (NMC). The purpose of this update is to publish the available evidence on breast cancer screening and provide information for decision-making in women at different levels of risk for developing the disease.

## METHODOLOGY

Searches were carried out in the Medline (PubMed), Excerpta Medica, Cochrane Library, Ebsco, Cumulative Index to Nursing and Allied Health Literature, and Latin-American and Caribbean Health Sciences Literature (via the Brazilian Regional Library of Medicine) databases, using as many keywords, descriptors and terms from the National Library of Medicine Medical Subject Headings list as possible, in order to collect scientific evidence on breast cancer screening with mammography, ultrasound, magnetic resonance imaging (MRI), and tomosynthesis, in women at average, intermediate, and high risk for breast cancer. We limited our searches to articles published between January 2012 and July 2022, in Portuguese, English, French, or Spanish. Complementary searches were carried out on websites and with online tools, as well as by hand searches of the bibliographies of the studies evaluated. The most recent, highest quality evidence (systematic reviews and meta-analyses) and the evidence that best answered the structured questions were selected for analysis. In their absence, primary studies (clinical trials or cohort studies) were included. The risk of bias in the studies was assessed by using the following: the Risk of Bias in Systematic Reviews tool; the Cochrane Risk of Bias Tools for Randomized Controlled Trials, version 2.0; the Quality Assessment of Diagnostic Accuracy Studies-Comparative tool; and the Risk of Bias in Non-randomized Studies-of Interventions tool. The overall quality of the evidence pool for each outcome was assessed by using the Grading of Recommendations Assessment, Development and Evaluation approach. Each recommendation was based on that evidence, through consensus of the joint committee of experts from the three entities (CBR, SBM, and FEBRASGO), defined as agreement of at least 75% of the committee members. If there was no initial agreement, a second round of discussion and voting was held, in which only a simple majority was needed. The recommendations for the various practices were classified into five categories:

**• Category A** - Recommendation **strongly in favor**, based on **high-quality** evidence
**• Category B** - Recommendation **strongly in favor**, based on **moderate-quality** evidence
**• Category C** - Recommendation **weakly in favor**, based on **low-quality** evidence
**• Category D** - Recommendation **in favor**, based on **expert consensus** only
**• Category E** - Recommendation **against**, because there is insufficient evidence to support its use

## RECOMMENDATIONS FOR SCREENING

### Screening of women at average risk

**■** MAMMOGRAPHY

- Annual mammographic screening, preferably with digital technology, is recommended for women between 40 and 74 years of age (*category A*).

- For women ≥ 75 years of age, it is recommended that screening continue if there are no comorbidities that reduce life expectancy and if the woman in question is expected to live for at least seven more years (*category D*).

**■** ULTRASOUND

- Ultrasound is not recommended as a supplementary screening test or as a stand-alone screening method for women at average risk (*category E*).

Note: *The use of ultrasound can be considered in specific higher-risk scenarios (see section on dense breasts, intermediate risk, and high risk)*.

**■** MRI

- MRI is not recommended as a supplementary screening test or as a stand-alone screening method for women at average risk (*category E*).

Note: *The use of MRI can be considered in specific higher-risk scenarios (see section on dense breasts, intermediate risk, and high risk)*.

**■** TOMOSYNTHESIS

- It is recommended that screening with tomosynthesis in combination with synthesized 2D (s2D) mammography or standard 2D mammography be considered when available (*category B*).

### Screening of women with dense breasts

**■** MAMMOGRAPHY

- Annual mammographic screening, preferably with digital technology, is recommended for women between 40 and 74 years of age with dense breasts (*category A*).

- For women ≥ 75 years of age with dense breasts, it is recommended that screening continue if there are no comorbidities that reduce life expectancy and if the woman in question is expected to live for at least seven more years (*category D*).

**■** ULTRASOUND

- It is recommended that annual ultrasound be considered as an adjunct to mammography in women with dense breasts, except when MRI is performed (*category B*).

**■** MRI

- It is recommended that biennial MRI be considered as an adjunct to mammography in extremely dense breasts (*category C*).

**■** TOMOSYNTHESIS

- It is recommended that screening with tomosynthesis in combination with s2D mammography or standard 2D mammography be considered when available (*category B*).

### Screening of women with a personal history of biopsy-proven atypical lobular hyperplasia, classical lobular carcinoma in situ, or atypical ductal hyperplasia

**■** INITIAL NOTE: It is recommended that women with a personal history of atypical lobular hyperplasia (ALH), classical lobular carcinoma in situ (LCIS), or atypical ductal hyperplasia (ADH) be evaluated by risk calculation models that include those variables in conjunction with other clinical data, including family history and breast density, to estimate their breast cancer risk.

**■** MAMMOGRAPHY

- For women diagnosed with ALH, LCIS, or ADH and an estimated lifetime risk < 20%, annual mammography is recommended from the age of 40 (*category A*).

- For women diagnosed with ALH, LCIS, or ADH and with an estimated lifetime risk ≥ 20%, annual mammography is recommended from diagnosis but no earlier than age 30 (*category B*).

**■** ULTRASOUND

- For women diagnosed with ALH, LCIS, or ADH and an estimated lifetime risk of 15-20%, ultrasound could be considered as an adjunct to mammography (*category D*).

- For women diagnosed with ALH, LCIS, or ADH and an estimated lifetime risk ≥ 20%, ultrasound is recommended as an alternative method for those who cannot undergo MRI, for whatever reason (*category B*).

**■** MRI

- For women diagnosed with ALH, LCIS, or ADH and with estimated lifetime risk ≥ 20%, annual MRI should be considered as an adjunct to mammography from diagnosis but no earlier than age 25 (*category B*).

**■** TOMOSYNTHESIS

- It is recommended that screening with tomosynthesis in combination with s2D mammography or standard 2D mammography be considered when available (*category B*).

### Screening of women with a personal history of treatment for invasive breast cancer or ductal carcinoma in situ

**■** MAMMOGRAPHY

- Women treated with **conservative surgery** for invasive breast cancer or ductal carcinoma in situ should undergo annual mammography (*category A*), starting at least six months after the end of radiotherapy.

- Women diagnosed with invasive breast cancer or ductal carcinoma in situ and treated with **mastectomy** should undergo annual mammography only of the contralateral breast, starting one year after the end of treatment (*category A*).

- For women diagnosed with invasive breast cancer or ductal carcinoma in situ who have undergone **nipple-sparing mastectomy**, mammography can be considered within the first year after the procedure to assess residual fibroglandular tissue, in order to determine the need for continued mammographic screening (*category D*).

**■** ULTRASOUND

- Ultrasound can be used as a complement to mammographic screening when MRI is indicated but cannot be performed, for whatever reason (*category C*).

**■** MRI

- Women who were treated with **conservative surgery** or **mastectomy** and were diagnosed with breast cancer before age 50 or have dense breasts should undergo annual MRI to evaluate the contralateral breast (*category C*), starting at one year after the end of treatment.

**■** TOMOSYNTHESIS

- It is recommended that screening with tomosynthesis in combination with s2D mammography or standard 2D mammography be considered when available (*category B*).

### Screening of women with a personal history of thoracic radiotherapy

**■** MAMMOGRAPHY

- Women with a personal history of thoracic radiotherapy before the age of 30 should undergo **annual mammography from the eighth year after radiotherapy but not before the age of 30** (*category A*).

**■** ULTRASOUND

- For women with a personal history of thoracic radiotherapy before the age 30, ultrasound should be used for screening only when MRI cannot be performed, for whatever reason (*category B*).

**■** MRI

- Women with a personal history of thoracic radiotherapy before the age of 30 should undergo **annual MRI from the eighth year after radiotherapy but not before the age of 25** (*category A*).

**■** TOMOSYNTHESIS

- It is recommended that screening with tomosynthesis in combination with s2D mammography or standard 2D mammography be considered when available (*category B*).

### Screening of women with a genetic mutation or with a strong family history of breast cancer (lifetime risk ≥ 20%)

**■** MAMMOGRAPHY

- Women with a pathogenic mutation of the *BRCA1* gene or not tested but with first-degree relatives who are carriers of such a mutation should undergo **annual mammography from the diagnosis of the mutation but not before the age of 35** (*category A*).

- Women with a pathogenic mutation of the *TP53* gene or not tested but with first-degree relatives who carry it should undergo **annual mammography from the diagnosis of the mutation but not before the age of 30** (*category A*).

- Women with a pathogenic mutation of the *BRCA2* gene or other genes who are at intermediate or high risk for breast cancer, as well as those who have not been tested but have first-degree relatives who are carriers of such mutations should undergo **annual mammography from the diagnosis of the mutation but not before the age of 30** (*category A*).

- Women with a lifetime risk ≥ 20%, calculated by one of the mathematical models based on family history, should undergo **annual mammography starting 10 years before the age at diagnosis of the relative who had been diagnosed at the youngest age, but not before the age of 30** (*category A*).

**■** ULTRASOUND

- Ultrasound should be used for screening only when MRI cannot be performed, for whatever reason (*category B*).

**■** MRI

- Women with pathogenic mutation of the *BRCA1* gene or not tested but with first-degree relatives who are carriers should undergo **annual MRI from the diagnosis of the mutation but not before the age of 25** (*category A*).

- Women with pathogenic mutation of the *TP53* gene or not tested but with first-degree relatives who are carriers, should undergo **annual MRI from the diagnosis of the mutation but not before the age of 20** (*category A*).

- Women with a pathogenic mutation of the *BRCA2* gene or other genes who are at moderate or high risk for breast cancer, as well as those who have not been tested but have first-degree relatives who are carriers, should undergo **annual MRI after the diagnosis of the mutation but not before the age of 30** (*category A*).

- Women with a lifetime risk ≥ 20%, calculated by one of the mathematical models based on family history, should have **annual MRI starting 10 years before the age at diagnosis of the relative who had been diagnosed at the youngest age, but not before the age of 30** (*category A*).

**■** TOMOSYNTHESIS

- It is recommended that screening with tomosynthesis in combination with s2D mammography or standard 2D mammography be considered when available (*category B*).

## JUSTIFICATION

The benefits of mammographic screening have been evaluated in cohort studies, systematic reviews, and randomized clinical trials, those studies having demonstrated that such screening provides a 22-30% reduction in breast cancer-specific mortality in women between 40 and 74 years of age^(2-4,7)^. When other important outcomes were analyzed among women who had undergone mammographic screening, better quality of life (measured in quality-adjusted life-years) was also observed, as a result of less aggressive treatments^(2)^, as was a higher rate of early-stage diagnosis of tumors, with better prognostic characteristics and node negative status^(3)^, as well as 28% fewer tumors diagnosed at an advanced stage^(4)^.

### Starting age and frequency of screening

Starting screening at age 40 reduces 10-year mortality from breast cancer by 25% but increases the false-positive (FP) rate from 4.8% to 7.0%^(7)^. According to data from the AMAZONA study^(8)^, 41.1% of women diagnosed with breast cancer in Brazil are under 50 years of age. As for the screening interval, it is noted that the biennial screening (in comparison with annual screening) is associated with a higher risk of advanced tumors (RR = 1.28), tumors larger than 15 mm, and tumors with worse prognostic factors^(7)^. Therefore, the NMC recommends annual mammographic screening from the age of 40.

### Considerations for women under 40

Screening for breast cancer is not recommended for women under 40, because of the lower incidence of breast cancer in this age group, which accounts for only approximately 7% of all cases. However, the AMAZONA III study showed that proportion to be 17% in Brazil, as well as that the tumors were larger and the prognosis at diagnosis was poorer among women under 40 than among women over 40^(9)^. Therefore, in agreement with other international societies^(10,11)^, the NMC recommends that the attending physician carry out an assessment of the estimated risk of breast cancer, using mathematical models, for all women over 30 years of age, in order to identify those at increased risk who could benefit from tailored screening.

### When to stop screening

Prospective studies and randomized controlled trials have not included women over 74 years of age, and there are therefore no direct data on screening in that age group. However, the life expectancy of women has increased, as has the incidence of breast cancer in the over-75 age group. Currently, 26% of breast cancer deaths occur in women diagnosed after the age of 74^(12,13)^. Considering these factors, many medical organizations recommend individualizing the decision, which should be discussed with the woman in question.

### Adverse effects of screening

Although some adverse effects of breast cancer screening have been reported, the quality of evidence for their analysis is low. Overdiagnosis is one effect that has been discussed, although its estimated magnitude varies because of the difficulty of determining whether a given tumor would or would not lead to the death of the patient^(14)^. The risk of carcinoma induced by the radiation used in mammographic screening is low, although it is higher in women with large breasts, in whom the radiation dose is higher, and in those submitted to extra views^(15)^. Breast cancer screening has also been associated with a 2.9% increase in the risk of biopsies with a benign outcome (FP), which can generate anxiety^(14)^. However, the reduction in mortality resulting from the early detection of cancer through screening outweighs the risks of damage caused by radiation exposure.

### Considerations regarding breast tomosynthesis

Tomosynthesis is an evolved form of digital mammography. Numerous studies confirm the effectiveness of this technology in breast cancer screening, which increases the detection rate by up to 50%^(16-20)^, as well as reducing the recall rate for additional imaging by 9-29%^(19,20)^. Tumors detected by tomosynthesis have histological and immunohistochemical characteristics similar to those of tumors detected by mammography^(21-23)^, and those results are maintained in subsequent rounds of screening^(24)^. Therefore, tomosynthesis, when accessible and available, is a screening method recommended by the NMC and other medical societies, including the American College of Radiology^(10)^, the American Cancer Society^(25)^, the European Society of Breast Imaging^(26)^, the Women’s Imaging Society of France^(27)^, and the National Comprehensive Cancer Network^(11)^, as well as in the European Guidelines on Breast Cancer Screening and Diagnosis^(28)^.

Tomosynthesis should be used in combination with standard 2D mammography or s2D mammography, the latter having the advantage of reducing the radiation dose^(15,17,18)^. Because the Brazilian Health Regulatory Agency has not yet established the reference and tolerance levels of the glandular dose for tomosynthesis, the recommendation is that each facility carry out a survey of the average glandular doses, using a sample of patients with breasts of different thicknesses, establishing local values for reference and tolerance levels^(29,30)^.

### Screening considerations for women with dense breasts

A dense breast is a risk factor for breast cancer and is associated with reduced sensitivity of mammography. Therefore, a number of supplementary methods have been proposed. Combining mammography with any of the proposed supplementary methods increases the sensitivity of the screening, allowing the identification of early-stage cancer that would be undetectable by mammography alone^(31-38)^.

MRI is the supplementary method with the highest rate of additional cancer detection^(31)^. That increases the likelihood of treatments that are less invasive and are curative. Data on critical outcomes such as mortality are not available. However, randomized trials have shown that the supplemental use of ultrasound in dense breasts or of MRI in extremely dense breasts reduces the rate of interval cancer, an important surrogate endpoint for patient-centered outcomes^(24,34,39)^. However, the use of supplementary methods is associated with an increase in the number of FPs and biopsies^(31,33,35-38)^. Nevertheless, for women with dense breasts without other risk factors, the NMC recommends screening with annual mammography starting at age 40, with the option of using supplementary methods such as ultrasound or MRI. For extremely dense breasts, there is scientific evidence suggesting that MRI is superior to the other supplementary methods.

### Screening considerations for women with a personal history of ALH, LCIS, or ADH

It has been demonstrated that ADH, ALH, and LCIS, which are considered non-obligate precursor lesions for ductal carcinoma in situ and invasive carcinoma^(40)^, confer an increased relative risk for the subsequent development of such carcinoma, that risk being 2.6-5.0 times greater for individuals with ADH, 3.2-4.8 times greater for those with ALH, and 6.0-10.0 times greater for those with LCIS^(41-49)^.

Studies evaluating breast cancer screening in women with a personal history of ADH, ALH, or LCIS are few and are based on retrospective series that estimated the risk for the subsequent development of in situ and invasive carcinomas. The current strategy to define screening in this subgroup is based on the calculation of the lifetime risk of breast cancer^(11)^. Factors such as age at diagnosis and breast density have a direct impact on breast cancer risk, which can be estimated using risk calculation tools based on mathematical models^(47)^. Currently, there are only a few models that include women with a personal history of ADH, ALH, or LCIS in the risk calculation. Such models include the Breast Cancer Risk Assessment Tool (Gail model) and the IBIS Breast Cancer Risk Evaluation Tool (Tyrer-Cuzizk model); these should preferably be used^(11,47)^.

### Screening considerations for women previously treated for invasive breast cancer or ductal carcinoma in situ

Women with a personal history of breast cancer have a seven times greater risk of developing a second malignant neoplasm in the ipsilateral or contralateral breast^(48)^. In those who have been treated with conservative surgery, mammography is less sensitive because of surgical alterations and a higher incidence of interval carcinoma^(49)^, which justifies the need for additional screening.

The use of MRI as a complement to mammographic screening can increase the detection rate by 8.2-18.1 cancers per 1,000 women^(50-55)^. The performance of MRI in that scenario has been shown to be similar to its performance in patients at high genetic risk, when the sensitivity, detection rate, FP rate, and positive predictive value of biopsies are taken into consideration^(56-58)^. However, the scientific evidence for the use of MRI in that population is weak, based predominantly on retrospective studies^(49,50,55-59)^. Within this heterogeneous group, the benefit of MRI is more well established in young patients (< 50 years of age at diagnosis) and in patients with dense breasts^(49-52)^.

Few studies have evaluated the accuracy of ultrasound, which has a detection rate of 2.4-4.3 additional cancers per 1,000 women over mammography, albeit with an increase in the FP rate and a lower positive predictive value for biopsies. When performed in addition to MRI, ultrasound does not improve sensitivity^(53,54)^, although it can be used as a supplemental screening method when MRI is not available.

In patients with a personal history of breast cancer treated with mastectomy, imaging-based screening of the treated breast, with or without reconstruction, is not indicated, because of the low rate of detection of asymptomatic cancers by mammography, ultrasound, or MRI in such patients^(59)^.

### Screening considerations for women with a history of thoracic radiotherapy

Women who undergo thoracic radiotherapy before age 30 have an average risk of developing breast cancer 13.4 times greater than that of the general population, similar to that of those with a mutation in the BRCA1 gene^(60)^. The increase in incidence occurs approximately 10 years after treatment and persists 30 years after. As previously shown^(61)^, the incidence rates are highest when patients are treated between 10 and 14 years of age (RR = 22.0) or between 15 and 19 years of age (RR = 14.3). For this group, there is evidence of the importance of screening with mammography and MRI, starting from the age of 25 or eight years after radiotherapy, in accordance with the recommendations of other medical entities, such as the Children’s Oncology Group and the International Guideline Group^(60)^.

### Screening of women with a genetic mutation or with a strong family history of breast cancer (lifetime risk ≥ 20%)

According to various studies^(62-64)^, mutations in genes that predispose to breast cancer are classified as high risk when they increase the risk by five times or more (e.g., mutations in the BRCA1, BRCA2, TP53, and PTEN genes) and as intermediate risk when they increase the risk by 1.5-5.0 times (e.g., mutations in the ATM, CHEK2, and BARD1 genes). In a study conducted in Brazil^(64)^, the most commonly mutated genes were found to be BRCA1 (in 27.4%), BRCA*2* (in 20.3%), TP53 (in 10.5%), ATM (in 8.8%), CHEK2 (in 6.2%), and PALB2 (in 5.1%). In that study, the Brazilian variant TP53 R337H was strongly associated with breast cancer risk (OR = 17.4). In the case of women with a strong family history of breast cancer but without a known mutation, those with an estimated **≥ 20% lifetime risk calculated** by mathematical models were categorized as being at high risk^(62)^. Such women develop cancer at an early age, with an incidence peak at 20-35 years of age for the PT53 mutation, at 30-39 years of age for the BRCA1 mutation, and at 30-49 years of age for the BRCA2 mutations, as well as at 40-59 years for women with high familial risk^(62-65)^.

For women with a strong family history of breast cancer, there is robust scientific evidence of the importance of MRI screening, because of the reduction in the rate of interval cancer and the higher detection rate for early-stage tumors, which can reduce the need for chemotherapy and reduce mortality, despite the increase in the number of FPs^(54,55,65-67)^. The role of mammography in patients with a BRCA1 mutation has recently been questioned. One meta-analysis^(68)^ demonstrated that the addition of mammography to MRI in patients with a BRCA1 mutation resulted in a modest (3.99%) increase in sensitivity and a similar (4.0%) reduction in specificity. For the BRCA2 mutation, the increase in sensitivity was greater (12.6%), with a small (5.0%) reduction in specificity. Therefore, the NMC recommends screening with MRI, together with mammography, but not starting mammography before 35 years of age for a BRCA*1* mutation or before 30 years of age for any other mutation. Additional ultrasound examinations do not yield additional detection of cancer if MRI is performed and should be reserved for further evaluation or to guide biopsies of findings identified on MRI.

As for the impact on mortality, one important study was conducted by Bae et al.^(54)^, which, despite being retrospective, demonstrated that high-risk women who underwent screening with mammography and MRI had better overall survival and tumors diagnosed at stages with a better prognosis than did those who underwent screening with mammography alone.

## CONCLUSION

These guidelines provide consensus recommendations based on current data for breast cancer screening in Brazil, subdivided into sections according to the risk for developing breast cancer, from the approach for women at average risk, who account for approximately 80% of all patients diagnosed with breast cancer, to that for women at higher risk.

## References

[1] Brasil, Ministério da Saúde, Instituto Nacional de Câncer [homepage on the
Internet] (2022). Estimativa 2023: incidência de câncer no Brasil.

[2] Moshina N, Falk RS, Botteri E (2022). Quality of life among women with symptomatic, screen-detected,
and interval breast cancer, and for women without breast cancer: a
retrospective cross-sectional study from Norway. Qual Life Res.

[3] Canelo-Aybar C, Ferreira DS, Ballesteros M (2021). Benefits and harms of breast cancer mammography screening for
women at average risk of breast cancer: a systematic review for the European
Commission Initiative on Breast Cancer. J Med Screen.

[4] Puliti D, Bucchi L, Mancini S (2017). Advanced breast cancer rates in the epoch of service screening:
the 400,000 women cohort study from Italy. Eur J Cancer.

[5] Urban LABD, Schaefer MB, Duarte DL (2012). Recommendations of Colégio Brasileiro de Radiologia e
Diagnóstico por Imagem, Sociedade Brasileira de Mastologia, and
Federação Brasileira das Associações de
Ginecologia e Obstetrícia for imaging screening for breast
cancer. Radiol Bras.

[6] Urban LABD, Chala FC, Bauab SP (2017). Breast cancer screening: updated recommendations of the Brazilian
College of Radiology and Diagnostic Imaging, Brazilian Breast Disease
Society, and Brazilian Federation of Gynecological and Obstetrical
Associations. Radiol Bras.

[7] Miglioretti DL, Zhu W, Kerlikowske K, Breast Cancer Surveillance Consortium (2015). Breast tumor prognostic characteristics and biennial vs annual
mammography, age, and menopausal status. JAMA Oncol.

[8] Simon SD, Bines J, Werutsky G (2019). Characteristics and prognosis of stage I-III breast cancer
subtypes in Brazil: the AMAZONA retrospective cohort study. Breast.

[9] Franzoi MA, Rosa DD, Zaffaroni F (2019). Advanced stage at diagnosis and worse clinicopathologic features
in young women with breast cancer in Brazil: a subanalysis of the AMAZONA
III study (GBECAM 0115). J Glob Oncol.

[10] Monticciolo DL, Newell MS, Moy L (2023). Breast cancer screening for women at higher-than-average risk:
updated recommendations from the ACR. J Am Coll Radiol.

[11] National Comprehensive Cancer Network [homepage on the
Internet] (2022). Breast cancer screening and diagnosis.

[12] Walter LC, Schonberg MA. (2014). Screening mammography in older women: a review. JAMA.

[13] Lee CS, Lewin A, Reig B (2023). Women 75 years old or older: to screen or not to
screen?. Radiographics.

[14] Hendrick RE, Helvie MA. (2011). United States Preventive Services Task Force screening
mammography recommendations: science ignored. AJR Am J Roentgenol.

[15] Miglioretti DL, Lange J, van den Broek JJ (2016). Radiation-induced breast cancer incidence and mortality from
digital mammography screening: a modeling study. Ann Intern Med.

[16] Friedewald SM, Rafferty EA, Rose SL (2014). Breast cancer screening using tomosynthesis in combination with
digital mammography. JAMA.

[17] Heindel W, Weigel S, Gerß J (2022). Digital breast tomosynthesis plus synthesised mammography versus
digital screening mammography for the detection of invasive breast cancer
(TOSYMA): a multicentre, open-label, randomised, controlled, superiority
trial. Lancet Oncol.

[18] Alabousi M, Wadera A, Kashif Al-Ghita MK (2021). Performance of digital breast tomosynthesis, synthetic
mammography, and digital mammography in breast cancer screening: a
systematic review and meta-analysis. J Natl Cancer Inst.

[19] Conant EF, Talley MM, Parghi CR (2023). Mammographic screening in routine practice: multisite study of
digital breast tomosynthesis and digital mammography
screenings. Radiology.

[20] Lowry KP, Coley RY, Miglioretti DL (2020). Screening performance of digital breast tomosynthesis vs digital
mammography in community practice by patient age, screening round, and
breast density. JAMA Netw Open.

[21] Yun SJ, Ryu CW, Rhee SJ (2017). Benefit of adding digital breast tomosynthesis to digital
mammography for breast cancer screening focused on cancer characteristics: a
meta-analysis. Breast Cancer Res Treat.

[22] Hovda T, Holen ÅS, Lång K (2020). Interval and consecutive round breast cancer after digital breast
tomosynthesis and synthetic 2D mammography versus standard 2D digital
mammography in breast screen Norway. Radiology.

[23] Dang PA, Wang A, Senapati GM (2020). Comparing tumor characteristics and rates of breast cancers
detected by screening digital breast tomosynthesis and full-field digital
mammography. AJR Am J Roentgenol.

[24] Pattacini P, Nitrosi A, Rossi PG, RETomo Working Group (2022). A randomized trial comparing breast cancer incidence and interval
cancers after tomosynthesis plus mammography versus mammography
alone. Radiology.

[25] Oeffinger KC, Fontham ET, Etzioni R, American Cancer Society (2015). Breast cancer screening for women at average risk: 2015 guideline
update from the American Cancer Society. JAMA.

[26] Sardanelli F, Aase HS, Álvarez M (2017). Position paper on screening for breast cancer by the European
Society of Breast Imaging (EUSOBI) and 30 national breast radiology bodies
from Austria, Belgium, Bosnia and Herzegovina, Bulgaria, Croatia, Czech
Republic, Denmark, Estonia, Finland, France, Germany, Greece, Hungary,
Iceland, Ireland, Italy, Israel, Lithuania, Moldova, The Netherlands,
Norway, Poland, Portugal, Romania, Serbia, Slovakia, Spain, Sweden,
Switzerland and Turkey. Eur Radiol.

[27] Société d’Imagerie de la Femme [homepage on the
Internet] Préconisation de la SIFEM sur l’utilisation de la
tomosynthèse en France.

[28] European Commission [homepage on the Internet] Tomosynthesis vs. digital mammography.

[29] Brasil, Ministério da Saúde, Agência Nacional de Vigilância
Sanitária Instrução Normativa - IN N° 92, de 27 de maio de
2021.

[30] Damilakis J, Frija G, Brkljacic B, European Society of Radiology (2023). How to establish and use local diagnostic reference levels: an
ESR EuroSafe Imaging expert statement. Insights Imaging.

[31] Hadadi I, Rae W, Clarke J (2021). Diagnostic performance of adjunctive imaging modalities compared
to mammography alone in women with non-dense and dense breasts: a systematic
review and meta-analysis. Clin Breast Cancer.

[32] Phi XA, Tagliafico A, Houssami N (2018). Digital breast tomosynthesis for breast cancer screening and
diagnosis in women with dense breasts - a systematic review and
meta-analysis. BMC Cancer.

[33] Ohuchi N, Suzuki A, Sobue T (2016). Sensitivity and specificity of mammography and adjunctive
ultrasonography to screen for breast cancer in the Japan Strategic
Anti-cancer Randomized Trial (J-START): a randomised controlled
trial. Lancet.

[34] Harada-Shoji N, Suzuki A, Ishida T (2021). Evaluation of adjunctive ultrasonography for breast cancer
detection among women aged 40-49 years with varying breast density
undergoing screening mammography: a secondary analysis of a randomized
clinical trial. JAMA Netw Open.

[35] Brem RF, Tabár L, Duffy SW (2015). Assessing improvement in detection of breast cancer with
three-dimensional automated breast US in women with dense breast tissue: the
SomoInsight Study. Radiology.

[36] Wu T, Warren LJ. (2022). The added value of supplemental breast ultrasound screening for
women with dense breasts: a single center Canadian
experience. Can Assoc Radiol J.

[37] Rebolj M, Assi V, Brentnall A (2018). Addition of ultrasound to mammography in the case of dense breast
tissue: systematic review and meta-analysis. Br J Cancer.

[38] Weigert J, Steenbergen S. (2015). The connecticut experiments second year: ultrasound in the
screening of women with dense breasts. Breast J.

[39] Bakker MF, de Lange SV, Pijnappel RM, DENSE Trial Study Group (2019). Supplemental MRI screening for women with extremely dense breast
tissue. N Engl J Med.

[40] Lopez-Garcia MA, Geyer FC, Lacroix-Triki M (2010). Breast cancer precursors revisited: molecular features and
progression pathways. Histopathology.

[41] Hartmann LC, Radisky DC, Frost MH (2014). Understanding the premalignant potential of atypical hyperplasia
through its natural history: a longitudinal cohort study. Cancer Prev Res (Phila).

[42] Worsham MJ, Abrams J, Raju U (2007). Breast cancer incidence in a cohort of women with benign breast
disease from a multiethnic, primary health care population. Breast J.

[43] London SJ, Connolly JL, Schnitt SJ (1992). A prospective study of benign breast disease and the risk of
breast cancer. JAMA.

[44] Collins LC, Baer HJ, Tamimi RM (2006). The influence of family history on breast cancer risk in women
with biopsy-confirmed benign breast disease: results from the Nurses’ Health
Study. Cancer.

[45] Menes TS, Kerlikowske K, Lange J (2017). Subsequent breast cancer risk following diagnosis of atypical
ductal hyperplasia on needle biopsy. JAMA Oncol.

[46] Page DL, Kidd TE Jr, Dupont WD (1991). Lobular neoplasia of the breast: higher risk for subsequent
invasive cancer predicted by more extensive disease. Hum Pathol.

[47] Brentnall AR, Cuzick J. (2020). Risk models for breast cancer and their
validation. Stat Sci.

[48] National Cancer Institute [homepage on the Internet] SEER Cancer Statistics Review (CSR) 1975-2018.

[49] Houssami N, Abraham LA, Kerlikowske K (2013). Risk factors for second screen-detected or interval breast
cancers in women with a personal history of breast cancer participating in
mammography screening. Cancer Epidemiol Biomarkers Prev.

[50] Gweon HM, Cho N, Han W (2014). Breast MR imaging screening in women with a history of breast
conservation therapy. Radiology.

[51] Giess CS, Poole PS, Chikarmane SA (2015). Screening breast MRI in patients previously treated for breast
cancer: diagnostic yield for cancer and abnormal interpretation
rate. Acad Radiol.

[52] Cho N, Han W, Han BK (2017). Breast cancer screening with mammography plus ultrasonography or
magnetic resonance imaging in women 50 years or younger at diagnosis and
treated with breast conservation therapy. JAMA Oncol.

[53] Berg WA, Zhang Z, Lehrer D, ACRIN 6666 Investigators (2012). Detection of breast cancer with addition of annual screening
ultrasound or a single screening MRI to mammography in women with elevated
breast cancer risk. JAMA.

[54] Bae MS, Sung JS, Bernard-Davila B (2020). Survival outcomes of screening with breast MRI in women at
elevated risk of breast cancer. J Breast Imaging.

[55] Sippo DA, Burk KS, Mercaldo SF (2019). Performance of screening breast MRI across women with different
elevated breast cancer risk indications. Radiology.

[56] Lehman CD, Lee JM, DeMartini WB (2016). Screening MRI in women with a personal history of breast
cancer. J Natl Cancer Inst.

[57] Weinstock C, Campassi C, Goloubeva O (2015). Breast magnetic resonance imaging (MRI) surveillance in breast
cancer survivors. Springerplus.

[58] Wernli KJ, Ichikawa L, Kerlikowske K (2019). Surveillance breast MRI and mammography: comparison in women with
a personal history of breast cancer. Radiology.

[59] Smith D, Sepehr S, Karakatsanis A (2022). Yield of surveillance imaging after mastectomy with or without
reconstruction for patients with prior breast cancer: a systematic review
and meta-analysis. JAMA Netw Open.

[60] Mulder RL, Kremer LCM, Hudson MM, International Late Effects of Childhood Cancer Guideline
Harmonization Group (2013). Recommendations for breast cancer surveillance for female
survivors of childhood, adolescent, and young adult cancer given chest
radiation: a report from the International Late Effects of Childhood Cancer
Guideline Harmonization Group. Lancet Oncol.

[61] Swerdlow AJ, Cooke R, Bates A (2012). Breast cancer risk after supradiaphragmatic radiotherapy for
Hodgkin’s lymphoma in England and Wales: a National Cohort
Study. J Clin Oncol.

[62] Rijnsburger AJ, Obdeijn IM, Kaas R (2010). BRCA1-associated breast cancers present differently from
BRCA2-associated and familial cases: long-term follow-up of the Dutch MRISC
Screening Study. J Clin Oncol.

[63] National Comprehensive Cancer Network [homepage on the
Internet] (2023). Genetic/familial high-risk assessment: breast, ovarian, and
pancreatic.

[64] Guindalini RSC, Viana DV, Kitajima JPFW (2022). Detection of germline variants in Brazilian breast cancer
patients using multigene panel testing. Sci Rep.

[65] Frebourg T, Lagercrantz SB, Oliveira C, European Reference Network GENTURIS (2020). Guidelines for the Li-Fraumeni and heritable TP53-related cancer
syndromes. Eur J Hum Genet.

[66] Chiarelli AM, Blackmore KM, Muradali D (2020). Performance measures of magnetic resonance imaging plus
mammography in the High Risk Ontario Breast Screening
Program. J Natl Cancer Inst.

[67] Saadatmand S, Geuzinge HA, Rutgers EJT, FaMRIsc study group (2019). MRI versus mammography for breast cancer screening in women with
familial risk (FaMRIsc): a multicentre, randomised, controlled
trial. Lancet Oncol.

[68] Phi XA, Saadatmand S, De Bock GH (2016). Contribution of mammography to MRI screening in BRCA mutation
carriers by BRCA status and age: individual patient data
meta-analysis. Br J Cancer.

